# Hunting for right and left parietal hot spots using single-pulse TMS: modulation of visuospatial perception during line bisection judgment in the healthy brain

**DOI:** 10.3389/fpsyg.2014.01238

**Published:** 2014-10-31

**Authors:** Adriana Salatino, Marisa Poncini, Mark S. George, Raffaella Ricci

**Affiliations:** ^1^Dipartimento di Psicologia, Università di TorinoTorino, Italy; ^2^Brain Stimulation Lab and Center for Advanced Imaging Research, Medical University of South CarolinaCharleston, SC, USA

**Keywords:** visuospatial perception, parietal cortex, neglect, Landmark task, TMS

## Abstract

A series of studies have consistently reproduced left neglect-like bias on line length estimation tasks in healthy participants by applying transcranial magnetic stimulation (TMS) over the right posterior parietal cortex (PPC), while no significant changes have been reported when stimulating the left PPC. However, a notable inter-individual variability in the right parietal site where TMS modulates visuospatial perception can be observed, and no general agreement exists on how to identify the optimal parietal site of stimulation. In the present study, we propose a new site-finding TMS protocol to easily identify the optimum parietal location, or “hot spot,” where TMS may modulate visuospatial perception on a line length estimation task (the Landmark task). Single-pulse TMS at 115% of motor threshold was applied 150 ms after the visual stimulus onset over nine different sites of a 3 cm × 3 cm grid, centred over right or left PPC (P4 and P3 according to the 10–20 EEG system, respectively) in eight healthy participants. Stimulation of right PPC induced a significant left neglect-like bias, when the coil was applied over the most posterior and dorso-posterior sites. Unexpectedly, TMS over left PPC also produced left neglect-like bias. However, in this case significant effects were found when targeting the most anterior and dorso-anterior portions of the grid. These results are discussed in relation to recent findings on neural networks underlying spatial cognition. The hunting protocol we propose might offer an economical and easy-to-use tool to functionally identify the optimal parietal site where TMS can modulate visuospatial perception, in healthy subjects and possibly in post-stroke patients undergoing repetitive transcranial magnetic stimulation treatment.

## INTRODUCTION

A series of studies (see for example, Fierro et al., 2000, 2001, 2006; Pourtois et al., 2001; Ellison et al., 2004; Ricci et al., 2012) have consistently induced left neglect-like bias on line length estimation in healthy individuals by applying transcranial magnetic stimulation (TMS) over the right posterior parietal cortex (PPC), while no significant behavioral changes have been observed when stimulating the left PPC (Fierro et al., 2000; Pourtois et al., 2001). Although different procedures have been used to identify the parietal site of stimulation, the most frequently employed method consists in the use of anatomical skull landmarks, as defined by the international 10–20 EEG system (i.e., P3, P4 or P5, P6). However, this easy and inexpensive procedure does not take into account inter-individual variability in brain structural or functional anatomy and given the small distance between contiguous regions around the intraparietal sulcus, the use of anatomical landmarks for coil placement may easily lead to targeting functionally different parietal areas (Herwig et al., 2003; Ricci et al., 2012). Indeed, considerable differences in PPC scalp locations where TMS modulated visuospatial attention have been found (Fierro et al., 2000, 2001; Pourtois et al., 2001; Oliveri and Vallar, 2009; Ricci et al., 2012; see Sack, 2010 for a review) and only a few studies have used neuroimaging to reveal the specific stimulated areas (Fierro et al., 2001; Herwig et al., 2001, 2003; Bjoertomt et al., 2002; Ricci et al., 2012). Ashbridge et al. (1997) proposed, for the first time, a single-pulse TMS “hunting” procedure to functionally localize the parietal site of stimulation where TMS could modulate performance on a conjunction visual search task. A 3 cm × 3 cm grid was centered over P4. Stimulation was initially applied over the center and progressively delivered to the other spots, following a counterclockwise order. The “visual hot spot” was localized at the location where TMS significantly increased the participants’ Reaction Times. More recently, Oliver et al. (2009) proposed a high frequency repetitive transcranial magnetic stimulation (rTMS) hunting paradigm with a “miss-stay” “hit-shift” protocol. Starting at P4, the coil was moved following a spiral shaped trajectory until the site where the participant missed, for at least four consecutive times, a small contralateral gap (that could appear on the peripheral left or right side of a horizontal line) was reached. However, both the above hunting procedures use visuospatial tasks that are seldom employed in TMS or neuropsychological investigations of visuospatial awareness. To our knowledge, no previous studies have explored the possibility to functionally identify the parietal site of stimulation during the execution of the Landmark task (Milner et al., 1993; Bisiach et al., 1998; Fierro et al., 2000, 2001; Ricci et al., 2012). This task, requiring to estimate which of two segments of a pre-bisected line is the shortest or longest, represents a non-manual variant of the line bisection, which constitutes one of the most commonly used tests for the evaluation of attentional biases in both healthy and neurologically impaired individuals (Ricci et al., 2000, 2004, 2014; Ricci and Chatterjee, 2001; Pierce et al., 2003; Savazzi et al., 2007; Chieffi et al., 2014) Here we propose a new hunting procedure to easily identify the optimal PPC scalp location where TMS can effectively modulate visuospatial perception in healthy subjects during line length estimation. In this study, we use a single-pulse TMS protocol very similar to the one used by Fierro et al. (2001), who showed, for the first time, the ability to induce neglect-like bias on the Landmark task by single pulses delivered 150 ms after visual stimulus presentation over the right PPC. A second aim is to explore the contribution of left PPC to visuospatial processes involved in the Landmark task, by applying the hunting protocol over left PPC, since only a few TMS studies have tested its role in the healthy brain.

## MATERIALS AND METHODS

Eight right-handed healthy participants (five women; mean age 24.25 years, range 21–28) with normal vision and no history of neurological or psychiatric illness, participated in the study. Participants were screened against inclusion/exclusion criteria for a safety use of TMS (Rossi et al., 2009) and a physical exam was performed by a licensed physician. In particular, since the most well-known safety concern of TMS is a seizure, potential participants who had a history of epilepsy or intracranial abnormality were not included. The study was performed at the Brain Stimulation Laboratory, Medical University of South Carolina. Participants were given a detailed explanation of the procedure, and they gave their written informed consent to participate in this study, which was approved by the Medical University of South Carolina Institutional Review Board.

The subjects’ visuospatial performance was evaluated on the Landmark Task, during both baseline conditions and during the application of single pulse-TMS over right and left PPC. The experimental apparatus consisted of a computer-assisted system able to deliver the TMS pulse time-locked to the visual stimulus.

### MAGNETIC STIMULATION

**FIGURE 1:**
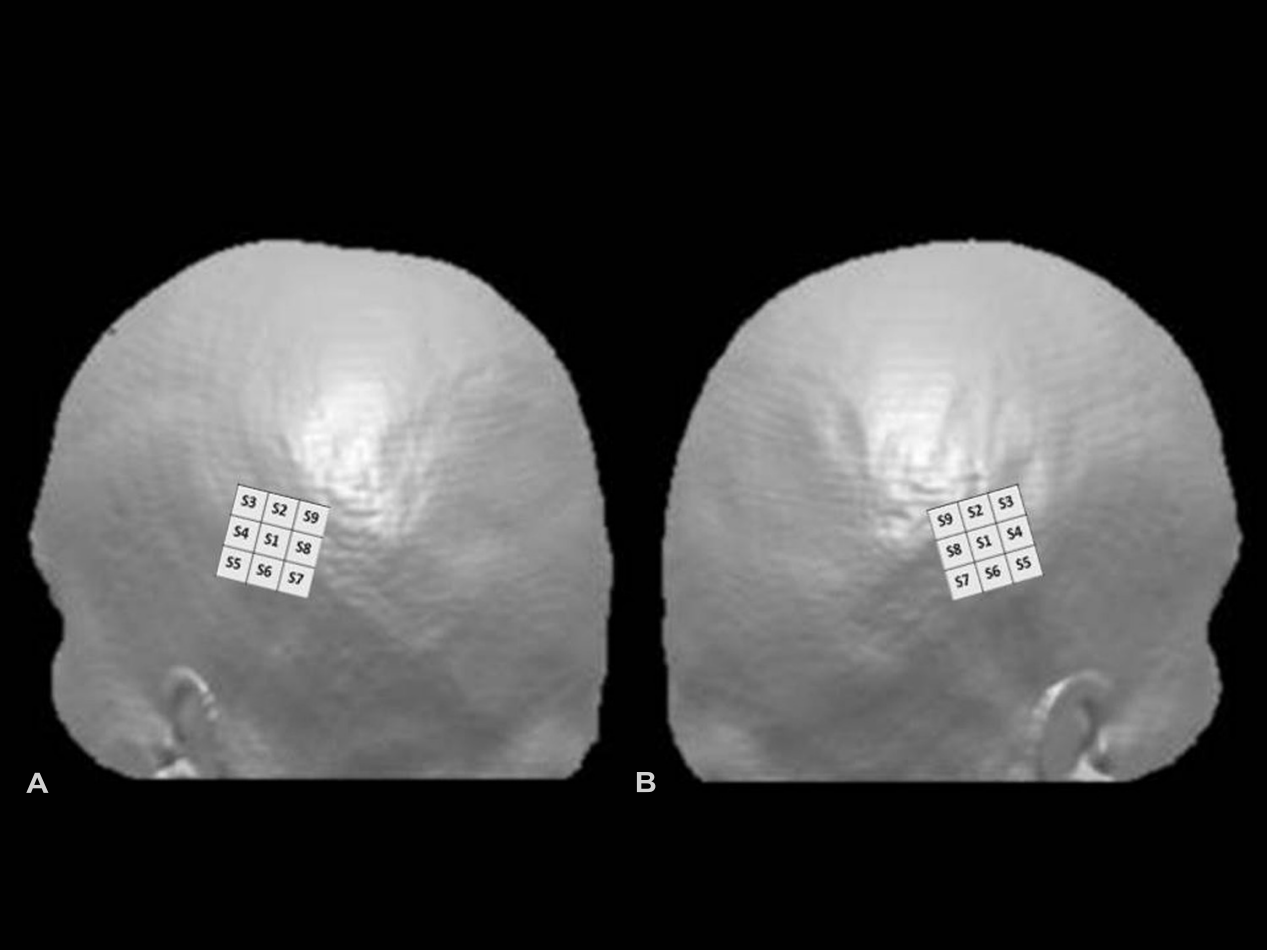
**Hunting grids for single-pulse TMS over left **(A)** and right **(B)** posterior parietal cortex (PPC).** Representation of the grid superimposed on 3D surface rendered MRI of the head using MRIcro (http://www.mricro.com, Rorden and Brett, 2000). In the figure, the 3 cm × 3 cm grid was centered over P3 (left PPC) or P4 (right PPC), according to the 10–20 EEG system. The S7–S8–S9 spots corresponded in both cases to the most posterior and S3–S4–S5 to the most anterior portions of the grid.

Transcranial magnetic stimulation was performed with a Neuronetics TMS system (Model 3600) with a solid focal coil (Neuronetics, Malvern, PA, USA). TMS was delivered over the right and left PPC in separate sessions. On the basis of previous studies (Ashbridge et al., 1997; Oliver et al., 2009), we used a 3 cm × 3 cm grid, centered over P4 or P3, according to the 10–20 EEG system. The grid was divided in nine spots, named S1–S9, with S1 as the spot at the center of the grid, S2 above it, and the other spots named following a clockwise order for the grid located over the right PPC and a counter-clockwise order for the grid located over the left PPC. In both cases, the S7–S8–S9 spots corresponded to the most posterior portion of the grid, and S3–S4–S5 to the most anterior sites (see **Figure 1**).

Single-pulses were delivered at an intensity of 115% of the subject’s resting motor threshold (rMT), 150 ms after the visual stimulus onset, as in previous single-pulse TMS studies showing induction of neglect-like bias on the Landmark task (Fierro et al., 2001; Ricci et al., 2012). The handle of the coil pointed backward and 45° downward from the parasagittal line. The inter-stimulus interval had duration of at least 4 s. Participants’ rMT was defined as the lowest stimulus intensity able to elicit a visible twitch in the abductor pollicis brevis muscle of the right hand in at least 5 of 10 consecutive stimulations of the motor hotspot. The participants were seated comfortably in front of a computer screen, which was centered on their sagittal mid-plane, at a distance of 60 cm from the screen. In order to provide *post hoc* projections of the site of stimulation where TMS significantly affected visuospatial performance, for two subjects (P1 and P3) MRI scans were co-registered with visible vitamin E on the subjects’ head and subsequently inspected using MRIcro (http://www.mricro.com, Rorden and Brett, 2000).

### VISUAL STIMULI

Visual stimuli consisted of white 0.09° of visual angle thick and about 20° of visual angle long horizontal lines, symmetrically transected by a 0.09° thick and 0.95° of visual angle high vertical bar, presented on the black screen of a computer monitor. Lines were presented at the center of the computer monitor for 50 ms. Before each stimulus presentation, a vertical line (0.95° of visual angle high) was presented for 500 ms at the center of the screen, to indicate the central fixation point. Subjects were required to verbally report which segment composing the pre-bisected line was shortest. They were asked to respond as quickly as they could, but not to sacrifice accuracy for speed. We asked participants to report the shortest segment of the line and did not ask them to perform the complementary task (i.e., to report the longest segment) employed in previous studies (Bisiach et al., 1998; Ricci et al., 2012) because we wanted to use a simplified version of the Landmark protocol and previous findings showed greater induction of perceptual rightward bias during the “shortest” than the “longest segment” task (Ricci et al., 2012). In the baseline condition, 10 visual stimuli were given without TMS in order to obtain the individual baseline. At the beginning of the stimulation procedure, the coil was placed on the central point of the grid (P4 or P3), which was the first site to be stimulated (S1 in the grid, see **Figure 1**). Then the coil was moved, in a clockwise direction, over the next point for right PPC condition, and in a counter-clockwise direction for left PPC condition (**Figures 1A,B**). Ten trials (10 visual stimuli accompanied by 10 single-pulses) were delivered for each spot, for a total of 90 trials. The same procedure was performed for the left and right parietal sites (P3 and P4). Between the two sessions, there was an interval of at least 30 min, to avoid possible residual TMS effects. For each hunting session (right and left PPC) a baseline condition was performed, and TMS trials of each session were referred to the baseline of the same session. The order for the left and right PPC conditions was balanced across subjects. To summarize, each subject underwent a total of 100 trials, 90 with TMS and 10 without TMS (baseline), for each parietal site.

The participants’ performance was evaluated by calculating, for each spot, the change in the direction of the response during TMS trials with respect to their baseline value. The percentage differences were analyzed both at individual and group level, for right and left hunting, using a test for differences between proportions (Bruning and Kintz, 1977).

## RESULTS

**Table 1** reports percentage values of right choices for baseline conditions, before TMS over right or left PPC. Participants reported the right segment as shortest most of the time.

**Table 1:** Individual and group performances on the Landmark task during baseline conditions.

Baseline	P1	P2	P3	P4	P5	P6	P7	P8	Mean
Right PPC (%)	60	60	90	80	70	80	60	70	71.25
Left PPC (%)	70	60	90	70	70	80	60	70	71.25

*Percentage values of right choices as shortest segment are reported for the baseline condition preceding TMS over right PPC and separately for the baseline condition preceding TMS over left PPC.*

**FIGURE 2:**
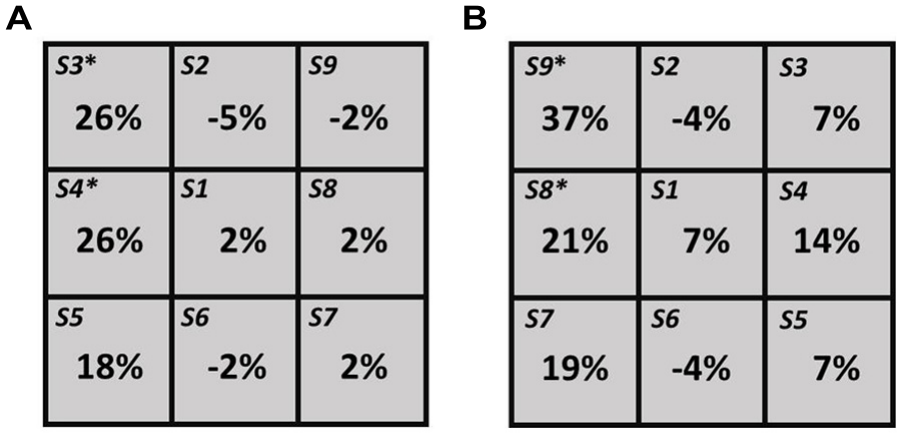
**Group-level results for left PPC **(A)** and right PPC **(B)** hunting conditions.** For each spot the values indicate the difference in percentage from the baseline value. The asterisk (*) indicates significant (*p* = 0.05) differences.

For the right hemisphere TMS condition, individual level analyses revealed a significant (*p* = 0.05, test of proportion, Bruning and Kintz, 1977) decrease in the number of times the right segment was perceived as shortest (i.e., left neglect-like bias) in three participants (P3, P5, and P8), when the TMS pulse was delivered in different posterior spots of the grid (S7, S8, and S9, see more details in **Table 2A**). One subject (P1) showed a significant (*p* = 0.05) bias in the opposite direction (i.e., increased number of times the right segment was chose as shorter) when the coil stimulated S1 (**Table 2A**). These findings reflect inter-individual variability of the right PPC site where stimulation can modulate visuospatial perception. At group level, a significant increase in the number of left choices as shortest segment (*p* = 0.05) was found in the two posterior spots S8 and S9 (**Figure 2B**).

**Table 2:** Individual performances on the Landmark task during right **(A)** and left **(B)** stimulation.

	S1	S2	S3	S4	S5	S6	S7	S8	S9
**(A) TMS over right PPC – individual analyses**
P1 (%)	-67*	-50	-33	0	0	-17	17	33	67
P2 (%)	50	-17	-17	-17	17	67	17	0	17
P3 (%)	33	0	11	44	33	0	44	22	89*
P4 (%)	-25	13	0	25	-13	-25	25	-13	-13
P5 (%)	29	0	43	14	0	0	-29	71*	0
P6 (%)	25	0	13	13	0	-25	13	-13	38
P7 (%)	17	17	33	50	17	0	-17	0	17
P8 (%)	-14	0	0	-29	0	-14	71*	71*	71*
**(B) TMS over left PPC – individual analyses**
P1 (%)	57	29	100*	100*	43	43	43	29	29
P2 (%)	0	-50	17	-17	-17	-67*	-17	0	-67*
P3 (%)	0	11	-11	33	44	11	0	0	0
P4 (%)	-29	-43	14	29	14	-43	-29	-14	14
P5 (%)	0	0	57	0	-14	57	29	14	14
P6 (%)	0	-13	38	38	25	-13	0	-25	0
P7 (%)	-17	33	17	17	33	-17	-33	0	33
P8 (%)	0	-14	-14	0	0	0	14	14	-43

**(A)** For each participant (P1–P8), performances on the Landmark task are reported, during the application of TMS over the nine spots of the grid (S1–S9) of the right PPC. The values indicate the relative percentage of difference from the baseline value. Positive values indicate rightward attentional bias (increased number of left choices as shortest segment) while negative values indicate leftward attentional bias (decreased number of left choices as shortest segment). *Significant at *p* < 0.05. **(B)** For each participant (P1–P8), performances on the Landmark task are reported, during the application of TMS over the nine spots of the grid (S1–S9) of the left PPC. The values indicate the relative percentage of difference from the baseline value. Positive values indicate higher number of left choices as shortest segment and, vice versa, negative values indicate decreased number of left choices as shortest segment. In this condition, where side of stimulation and side of segment underestimation are congruent, a positive value may either indicate a perceptual left neglect-like bias as well as a leftward response bias (see the text for further details). *Significant at *p* < 0.05.

For the left hemisphere TMS condition, individual level analyses revealed a significant (*p* = 0.05) increase of left choices in one participant (P1), when TMS was delivered in two anterior spots of the grid (S3 and S4, **Table 2B**). The opposite bias (*p* = 0.05) was observed in one subject (P2), when targeting S6 and S9 (**Table 2B**). At group level, a significant increase of left choices (*p* = 0.05) was found when TMS was delivered over S3 and S4 (see **Figure 2A**).

*Post hoc* projections showed that for one participant (P3) one of the right PPC sites where TMS induced left neglect-like bias (i.e., S9) overlaid the right angular gyrus (AG; approximate MNI coordinates: 55, -66, 39). In another representative participant (P1), *post hoc* projections showed that one of the left PPC spots where TMS modulated visuospatial perception (i.e., S3) overlaid the left AG (approximate MNI coordinates: -48, -59, 38).

## DISCUSSION

With this study we propose an easy to use protocol to identify the best coil location over PPC where TMS modulates visuospatial perception during the execution of line length estimation in healthy participants. We chose the Landmark task because of its frequent use in studies of spatial cognition in both healthy individuals (Fierro et al., 2000, 2001, 2006; Fink et al., 2000, 2001; Çiçek et al., 2009; Pia et al., 2012; Ricci et al., 2012; Benwell et al., 2014) and patients with neglect (Bisiach et al., 1998, 1999a,b; Brighina et al., 2003). Single-pulse TMS was intermittently and time-locked applied over right and left PPC. A nine points grid was centred over P4 or P3 and stimuli were delivered at each location (S1–S9).

As expected, participants showed pseudoneglect (i.e., right segment underestimation) during baseline conditions (i.e., without TMS). Individual level analyses revealed inter-individual variability of the right and left PPC sites where stimulation modulated line-length judgments. This outcome might be due to anatomical and functional inter-individual differences. In some participants the lack of effects by TMS might be explained by a low degree of right hemisphere lateralization of the visuospatial system. In support of this hypothesis is the finding that two (P3, P8) out of three participants who showed a statistically significant effect were males and therefore individuals who are more likely to present pronounced hemispheric asymmetries (Catani et al., 2007). Consistently with previous findings (Fierro et al., 2000, 2001; Pourtois et al., 2001; Ricci et al., 2012), participants, as a group, showed left neglect-like perceptual bias when TMS was applied over right PPC. Specifically, the effect was found when stimulating two sites located 1 cm posterior and 1 cm dorso-posterior to P4 (S8 and S9, respectively). These locations roughly correspond to P6, an electrode site successfully used in previous TMS studies inducing rightward spatial bias on the Landmark task in healthy participants (Fierro et al., 2000, 2001; Brighina et al., 2003). This finding is also consistent with neuroimaging evidence showing a significant involvement of right PPC in the healthy brain during visuospatial judgment on this task (Fink et al., 2000, 2001; Çiçek et al., 2009). Unexpectedly, an increase in the number of left choices as shortest segment was also found during stimulation of the left parietal cortex, when the coil was targeting the most dorso-anterior and anterior portions of the grid.

Involvement of right PPC in visuospatial processing and lack of results by stimulation of the left PPC have been documented in previous TMS studies (Ashbridge et al., 1997; Fierro et al., 2000; Sack et al., 2007) that, in line with neuroimaging evidence (Fink et al., 2000, 2001; Corbetta and Shulman, 2002; Siman-Tov et al., 2007; Çiçek et al., 2009; Thiebaut de Schotten et al., 2011), support the notion of right hemisphere dominance for visuospatial cognition. In previous TMS investigations (Ashbridge, et al., 1997; Fierro et al., 2000; Sack et al., 2007) only a spot was stimulated over left PPC. In contrast, here we applied TMS over P3 and its surrounding sites, searching for the location where stimulation could modulate the participants’ performance. Surprisingly, TMS over left PPC produced the same bias that was induced by TMS over right PPC. This finding can be explained by induction of a left neglect-like perceptual bias (i.e., left segment underestimation) via TMS inter-hemispheric effects. Indeed the effects of TMS over left PPC might have propagated to right PPC, inducing a similar behavioral outcome as shown during right hemisphere stimulation. This interpretation is in line with the physiology of the corpus callosum characterized by a predominance of excitatory inter-hemispheric connections and with recent findings demonstrating that low frequency rTMS or single-pulse TMS can induce bilateral decreases of cortical excitability (Wassermann et al., 1998; Nowak et al., 2008) or brain activity (Ricci et al., 2012), respectively. However, an increase of left segment choices during left hemisphere stimulation might also be explained by a leftward response bias (i.e., a tendency to respond toward the side of stimulation, see Ricci et al., 2012). Unfortunately, the exclusive use of the task requiring to choose the shortest segment does not allow one to disentangle the two alternative interpretations when stimulation is applied over left PPC, since side of stimulation and side of segment underestimation are congruent. We used only one of the two tasks employed in previous studies (i.e., choose the “shortest” vs. “longest” segment, Bisiach et al., 1998; Ricci et al., 2012) with the aim of proposing a short protocol that was able to induce the greatest effects (Ricci et al., 2012) and disambiguate perceptual and response bias when TMS was applied over the right PPC. However, given the effects of left hemisphere stimulation, the exclusive use of the “shortest” task constitutes a limitation. The inclusion of the complementary condition (the “longest” task) needs to be considered in future investigations aimed at exploring the contribution of left parietal cortex to visuospatial judgment on the Landmark task.

Neuroimaging results from two representative individuals indicate that the structure underlying right (S9) and left (S3) dorsal sites, where TMS induced a rightward bias, was in both cases the AG. The result that the AG was the stimulated area during TMS induction of a rightward bias on the Landmark task is consistent with previous evidence (Ricci et al., 2012). In this study, the interleaved TMS/fMRI technique revealed, during induction of the spatial bias, decreased neural activity of parieto-frontal networks, mainly in the right (stimulated) hemisphere, overlapping with regions directly linked through the SLFII (Thiebaut de Schotten et al., 2011). This tract, that originates from the AG and has a greater volume in the right than in the left hemisphere, seems to play a crucial role in visuospatial processing underlying line bisection (Thiebaut de Schotten et al., 2005, 2011; Bartolomeo et al., 2007).

A study limitation, beside the use of only one kind of task, is the small number of trials administered to each site of the nine points grid that likely prevented from finding statistical significance in the majority of individual analyses. Indeed, a higher number of trials would have increased the statistical power and thus the number of significant differences at individual level. Future investigations should consider to double the number of trials by administering the two complementary tasks and overcome the above issues. However, we advise to define a qualitative rather than quantitative criterion for the hunting protocol as in previous instances (Oliver et al., 2009), given the necessity to identify, quickly and efficiently, the useful site of stimulation. For the proposed hunting procedure we suggest to consider as best location the site with highest number of changes in the response direction with at least a difference of 3 out of 10 responses with respect to baseline condition.

In spite of its limitations, the current study adds important information about the parietal sites over which TMS may affect line length estimation. In addition, the finding of rightward spatial bias during stimulation of left PPC might shed some novel insight into the asymmetry of the neural mechanisms underlying spatial cognition. Future investigation is necessary to validate and further explore these findings.

The hunting protocol we propose might offer an economical and easy functional procedure to identify the optimal parietal site where TMS can modulate visuospatial perception, in healthy subjects and possibly in post-stroke patients undergoing rTMS treatment (Brighina et al., 2003; Salatino et al., 2014). Additionally, specific variants of this protocol may offer the possibility to investigate the differential contribution of diverse portions of right and left hemisphere PPC to bilateral networks for spatial cognition.

## Conflict of Interest Statement

The authors declare that the research was conducted in the absence of any commercial or financial relationships that could be construed as a potential conflict of interest.
